# Supplementary material for: Benefits of High-Flow Nasal Cannula Therapy for Acute Pulmonary Edema in Patients with Heart Failure in the Emergency Department: A Prospective Multi-Center Randomized Controlled Trial
Source: J Clin Med. 2020 Jun 21;9(6):1937. doi: 10.3390/jcm9061937 (PMC7355695; doi:10.3390/jcm9061937)
Supplement: Supplementary file 1 [file jcm-09-01937-s001.pdf]

Table S1. Reference ranges for results of laboratory test.

Laboratory Test	Reference Range
White blood cell count (μL)	4000~10800
Hemoglobin (g/dL)	13~17
Hematocrit (%)	40~52
Platelet count (10^3/μL)	150~400
Blood Urea Nitrogen (mg/dL)	8.6~23.0
Creatinine (mg/dL)	0.72~1.19
Albumin (g/dL)	3.4~5.3
Aspartate aminotransferase (IU/L)	16~37
Alkaline Phosphatase (IU/L)	11~46
Total bilirubin (mg/dL)	0.3~1.8
Sodium (mmol/L)	138~146
Potassium (mmol/L)	3.6~4.8
Chloride (mmol/L)	96~107
Creatine Kinase (U/L)	35~232
Creatine Kinase myocardial band isoenzyme (mcg/L)	0~6.73
Troponin-I (mcg/L)	0.0121~0.080
Pro-Brain Natriuretic Peptide (pg/mL)	0~738
